# Application of Sub-Micrometer Vibrations to Mitigate Bacterial Adhesion

**DOI:** 10.3390/jfb5010015

**Published:** 2014-03-11

**Authors:** Will R. Paces, Hal R. Holmes, Eli Vlaisavljevich, Katherine L. Snyder, Ee Lim Tan, Rupak M. Rajachar, Keat Ghee Ong

**Affiliations:** Department of Biomedical Engineering, Michigan Technological University, Houghton, MI 49931, USA; E-Mails: wrpaces@mtu.edu (W.R.P.); hrholmes@mtu.edu (H.R.H.); evlaisav@mtu.edu (E.V.); klsnyder@mtu.edu (K.L.S.); eltan@mtu.edu (E.L.T.)

**Keywords:** antifouling, magnetoelastic materials, sub-micron vibrations

## Abstract

As a prominent concern regarding implantable devices, eliminating the threat of opportunistic bacterial infection represents a significant benefit to both patient health and device function. Current treatment options focus on chemical approaches to negate bacterial adhesion, however, these methods are in some ways limited. The scope of this study was to assess the efficacy of a novel means of modulating bacterial adhesion through the application of vibrations using magnetoelastic materials. Magnetoelastic materials possess unique magnetostrictive property that can convert a magnetic field stimulus into a mechanical deformation. *In vitro* experiments demonstrated that vibrational loads generated by the magnetoelastic materials significantly reduced the number of adherent bacteria on samples exposed to *Escherichia coli*, *Staphylococcus epidermidis* and *Staphylococcus aureus* suspensions. These experiments demonstrate that vibrational loads from magnetoelastic materials can be used as a post-deployment activated means to deter bacterial adhesion and device infection.

## 1. Introduction

Opportunistic bacterial infection represents a principle danger in implantable biomedical devices, both in terms of the patient’s health and functionality of the implant [1,2,3]. The majority of these infections arise from colonization by members of the genus Staphylococcus, primarily *Staphylococcus aureus* and *Staphylococcus epidermidis*, as well as *Escherichia coli* [2]. As such, the mediation of these bacteria is paramount to improve device success. The Staphylococci are gram positive facultative aerobes that exist typically as a constituent of human skin flora [1]. While *S. aureus* tends to be more strongly pathogenic, *S. epidermidis* is more likely to adhere to and produce a biofilm on polymer surfaces, including those typically used as a component of many implants [4]. Conditions arising from Staphylococcus infections have become increasingly more common as anti-biotic resistant strains arise [5,6,7]. *E. coli* are gram negative bacteria that commonly reside within the human intestinal tract [8,9]. In addition to non-specific adhesion characteristics, *E. coli* exhibit FimH bonding sites that can mediate further complications in cases involving infections of implanted devices that are exposed to fluid shear stress [8,10]. As a result of allostery, FimH bonds can be strengthened under these conditions [8], thereby enhancing bacterial attachment and colonization. This binding region represents only one of the vast multitude of species-specific adhesion mechanisms apparent in the bacterial domain. As most bacteria must adhere to a surface in order to survive and propagate, virtually every species has developed multiple unique binding methods; these range from simple nonspecific interactions [2] and membrane proteins [11] to sophisticated cellular structures [10,12] and behaviors [10,12,13,14]. The variance inherent in these systems clarifies the somewhat limited efficacy of antibiotics in their use against preventing infection.

Antimicrobial control of bacteria is typically achieved in one of two ways: through either administration of antibiotics or the modification of a material’s inherent properties. Since their widespread introduction in the 1940’s, antibiotics have been used to mitigate bacterial infection [5,6,7]. While treatments that incorporate a drug-centered antibacterial regime have been met with general success, their efficacy has come into question with the emergence of antibiotic resistant bacteria [5,6,7,15,16]. Some material treatments, such as those that incorporate silver ions, have been shown to achieve this same effect without giving rise to resistant strains [17,18]. Silver anions absorbed by bacteria impede the replication process by interfering with RNA translation and enzyme function [18]. Although moderately effective, silver coated materials pose the limited threat of unintentionally delivering toxins to host cells and causing heavy metal buildup in vital organs [18]. As an alternative to incorporating cytotoxins, many antimicrobial materials prevent cellular adhesion to their surface through innate properties such as low surface roughness and charge [1,7,17]. For example, Parylene-C is a polymer that possesses intrinsic antimicrobial properties; it is widely inert, resists degradation, and maintains a uniform coating [19,20,21]. Additionally, Parylene-C can be highly functionalized; its hydrophobicity and roughness can be modified with exposure to oxygen plasma [22]. Despite its optimal surface properties, Parylene-C materials are still susceptible to bacterial incursion and infection. Although protein adsorption, and therefore cellular adhesion, can be somewhat deterred through material surface treatment, it is far from eliminated.

Although an effective measure for discouraging cellular adhesion, surface chemistry accounts for only an interim solution. Biofilm producing bacteria effectively colonize even microbe resistant surfaces as a result of an excreted glycoprotein matrix [1]. As an alternative to surface modification, mechanical stimuli have also been shown to modulate bacterial adhesion [23]. The effect of these mechanical stimuli are typically investigated by observing cultures under fluid shear stress, however, during mechanical loading via fluid flow, the magnitude of forces at a given surface reduce to zero. Similarly, ultrasonic acoustics have been shown to modulate biofilm formation when applied to a bacterial culture [23,24,25,26]. This treatment, however, must be coupled with an antibiotic in order to maintain efficacy and may be practical only for certain devices, such as catheters [25,26]. Magnetoelastic (ME) materials are a type of magnetostrictive material that converts a magnetic stimulus into a mechanical deformation. Considering the fact that a magnetic field can penetrate tissue with minimum attenuation, these materials can be activated externally following implantation; thereby providing a platform for non-invasive, post-deployment treatment. These materials have been used as sensors to measure pressure [27], cell activity [28,29] and a number of other physiological variables [30]. Furthermore, it has been recently demonstrated that the vibrations produced by ME materials can modulate cell adhesion [30]. Magnetic fields and materials have been employed as functional biomaterials [31,32,33]. For instance, nanocomposite magnetic scaffolds, consisting of poly (caprolactone) and superparamagnetic hydroxyapatite nanoparticles [32] or iron oxide [33], have been synthesized for bone tissue engineering.

The goal of this study was to develop a novel means of mitigating bacterial adhesion through vibrational loads generated with ME materials. We hypothesize that the sub-micrometer (sub-micron) vibrations generated by activated ME materials can reduce bacterial adhesion. To establish the propensity of using ME materials to generate vibrational loading, a free-standing strip of ME material was remotely activated by an alternate current (AC) magnetic field, exciting a vibration within the material at a characteristic response frequency that subjected adherent bacteria to sub-micron vibrations. 

## 2. Results and Discussion

**Figure 1:**
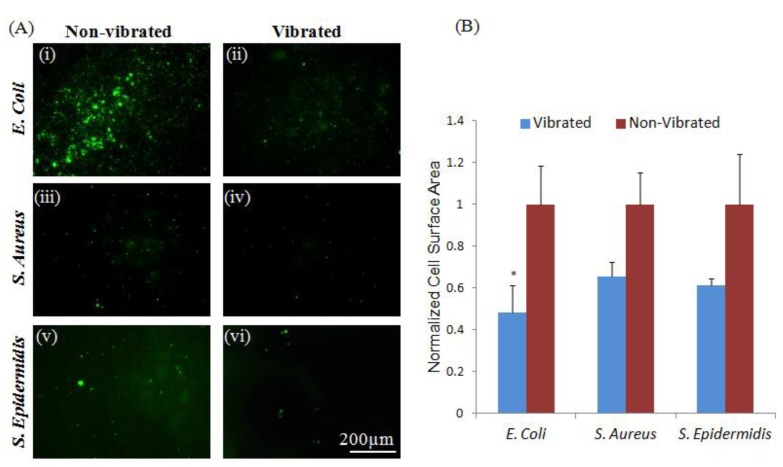
Vibrations from magnetoelastic (ME) materials can modulate bacterial adhesion to Parylene-C coated materials. (**A**) Vibrations induced a significant (*p* < 0.05) reduction in adhesion of *E. coli* (i, ii). No significant changes in the adhesion of *S. aureus* or *S. epidermidis* (iii–vi) were observed between loaded and unloaded groups; note that these strains were effectively non-adherent on control surfaces, possibly a result of the Parylene-C surface character; (**B**) Quantitative measurements were normalized to non-vibrated samples. Error bars represent ±standard error of the mean (S.E.M.).

The low surface energy associated with untreated Parylene-C [34] deters attachment of some bacterial strains, as evidenced by the low levels of adhesion apparent in both *S. epidermidis* and *S. aureus* (Figure 1). No significant (*p* < 0.05) difference was observed between control groups and samples exposed to sub-micron vibrations, as limited initial adhesion was insufficient to show a decrease in attachment. However, as adhesion mechanisms are species specific [11,12,13]. *E. coli* were shown to adhere to untreated Parylene-C coated ME materials despite its anti-microbial surface characteristics. Loading adherent *E. coli* with sub-micron vibrations produced by the ME material substrate resulted in a significant (*p* < 0.05) reduction in adherent bacteria. These results indicate that vibrational loads generated by the ME materials can be used as a secondary or supplementary means to mitigate bacterial adhesion. In situations where an antimicrobial or drug releasing surface has been compromised through degradation, ME materials can be implemented to generated vibrations to further prevent bacterial adhesion and device infection.

**Figure 2:**
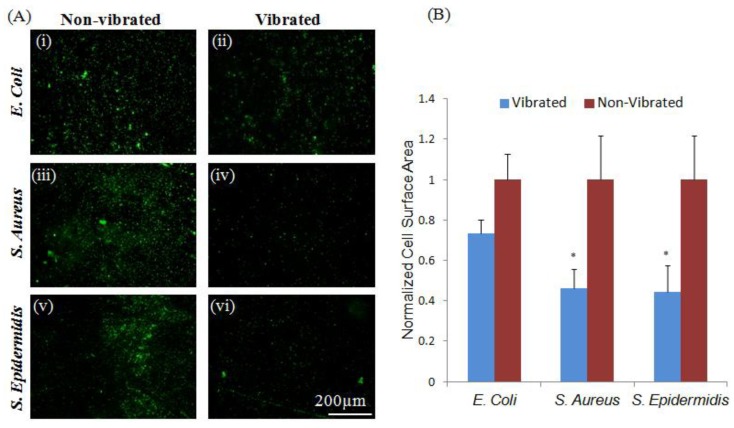
Bacteria adherent to plasma-etched Parylene-C coated materials show species specific response to sub-micron vibrations. (**A**) Vibrations did not induce significant (*p* < 0.05) changes in the adhesion of *E. coli* (i–ii). However, a significant reduction in the adhesion of *S. aureus* and *S. epidermidis* (iii–vi) was observed in response to sub-micron vibrations. Changes in surface character may be responsible for the increased adherence of *S. aureus* and *S. epidermidis* in plasma-treated controls, as adhesins produced by these strains are known to bind more readily to hydrophilic surfaces; (**B**) Quantitative measurements were normalized to non-vibrated samples. Error bars represent ±S.E.M.

It was further observed that etching Parylene-C coated ME materials with O_2_ plasma [35] produced a surface more conducive to adhesion of both Stapholycocci strains and *E. coli* (Figure 2). This difference in adhesive characteristic was likely the result of significant changes in hydrophobicity and roughness of the exposed surfaces from 84.2° and 9.01 respectively in Parylene-C coated samples to 21.3° and 26.3 following plasma treatment (Table 1). Additionally, these differences may also have been the result of modifications made to the Parylene-C surface [23]. The application of sub-micron vibrations to bacteria cultured on these surfaces resulted in significant (*p* < 0.05) decreases in the adhesion of both Staphylococcus strains; however, no significant change in the adhesion of *E. coli* was observed, perhaps as a result of advantageous species-specific binding character on this particular substrate [9,36]. This observation may suggest the existence of species-targeted vibrational treatments. Previous research has shown that varying intensities of acoustic vibration has a marked effect on the behavior of exposed bacteria; ranging from the eradication of biofilms to stimulating their production [24,25,26]. Slightly altering the vibrational amplitude may result in a differing bacterial response; further experimentation may produce practical species targeted treatment regimes. 

**Table 1:** Surface properties and chemical structure. Table denotes contact angle, surface roughness and surface energy of polymer coatings used. Monomer units of Parylene-C include benzene and chloride functional group constituents that impart resonant stability and inertness. Monomer units of Poly-L-Lactide (PLLA) include a ketone and carbonyl functional groups that can be exploited by some bacterial adhesins.

Surface	Contact Angle (°θ)	Surface Roughness (RMS Average)	Surface Energy (mJ/m^2^)	Chemical Structure
Parylene-C	84.2	9.01	19.6 [37]	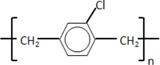
Plasma Etched Parylene-C	21.3	26.3	46.5 [38]	–
PLLA	71.7	0.211	43.2 [39]	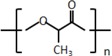

**Figure 3:**
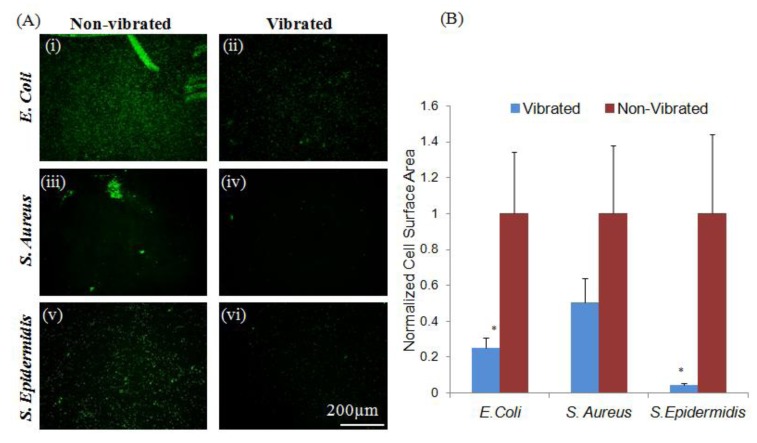
Sub-micron vibrations significantly reduce adhesion of bacteria to explanted Parylene-C coated materials. (**A**) Vibrations induced a significant (*p* < 0.05) reduction in adhesion of *E. coli* and *S. epidermidis* (i, ii; v, vi), however, sufficient levels of *S. aureus* attachment were not achieved on control samples to demonstrate significance (iii, iv); (**B**) Quantitative measurements were normalized to non-vibrated samples. Error bars represent ±S.E.M.

Following a 60-day implantation period, Parylene-C coated materials were observed to maintain varying levels of protein adsorption and tissue growth. Surface conditions acquired in this manner simulate an *in vivo* physiologically relevant environment. Although affected surfaces were observed to be more conducive to bacterial attachment than untreated Parylene-C, the overall surface of the explanted materials lacked uniformity. While materials subjected to both *E. coli* and *S. epidermidis* strains demonstrated relatively high levels of adhesion, an absence of adherent bacteria was observed in samples exposed to *S. aureus* cultures. Subjecting these materials to sub-micron vibrations resulted in a significant (*p* < 0.05) decrease in the number of adherent *E. coli* and *S. epidermidis* bacteria (Figure 3). No significant changes in the adhesion of *S. aureus* were observed as a result of competitive exclusion by deposited proteins [38], deterring initial bacterial adhesion. While these results indicate that sub-micron vibrational loading can be used both as a post-deployment means to mitigate device infection and are not limited to use only immediately after implantation, they also demonstrate that this treatment can be used effectively on widely different bacterial strains. Therefore, this treatment could be profoundly useful in a variety of clinical applications.

**Figure 4:**
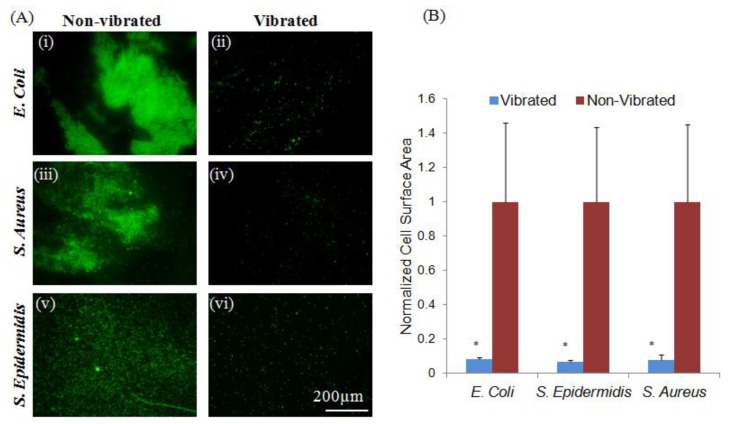
Sub-micron vibrations modulate bacterial adhesion to a chemically active surface. (**A**) Quantitative analysis of area covered by *E. coli*, *S. aureus* and *S. epidermidis* bacterial colonies shows significant (*p* < 0.05) reduction in adhesion to PLLA treated material surfaces when subjected to vibrations; (**B**) Quantitative measurements were normalized to non-vibrated samples. Error bars represent ±S.E.M.

The chemical composition of PLLA does not limit microbial adhesion to the same extent as Parylene-C [40]. As such, elevated levels of adhesion for all bacterial strains were observed on ME materials coated with PLLA (Figure 4). Although Parylene-C has been shown to exhibit greater roughness and hydrophobicity, the differences in surface energy between PLLA and etched Parylene-C coated materials are significant as a result of surface chemistry [1,37,39]. Bacterial adhesion of all strains to PLLA coated ME materials was shown to be significantly (*p* < 0.05) reduced by the application of sub-micron vibrations. This result suggests that sub-micron vibrations have the potential to serve not only as a secondary means of bacterial deterrence, but as a primary means as well. This treatment could be used in applications where a material surface must maintain some level of chemical activity for proper interface, or in cases where a surface treatment that deters wide-scale cellular attachment is impractical. Under these circumstances, a ‘race to the surface’ between host tissue cells and pathogenic bacteria occurs; once either gains a significant presence on the material, the other is generally rejected, leading either to device integration or infection [38]. Therefore, in cases where sub-micron vibrational loading can be applied to deter bacterial adhesion, tissue integration can occur more reliably and improve medical device stability.

## 3. Experimental Section

### 3.1. Preparation of Magnetoelastic (ME) Samples

Magnetoelastic materials were prepared for *in vitro* testing by mechanically shearing a ribbon (Metglas 2826MB Fe_40_Ni_38_Mo_4_B_18_, Metglas Inc., Conway, SC, USA) into strips with width 5 mm and length 12.8 mm (thickness = 26 μm) for all experiments. This material was chosen for its saturation magnetostriction (12 ppm) and magnetic relative permeability (>50,000) [41]. Materials were then sonicated in isopropyl alcohol for 180 s and annealed (125 °C for 2 h) to remove defects and alleviate internal stresses.

#### 3.1.1. Parylene-C Coating

Following annealing, materials were coated with a 10 μm thick Parylene-C layer via vapor deposition PDS 2010 LABCOTER^TM^ 2 (Specialty Coating Systems, Indianapolis, IN, USA) using manufacturer’s protocol to maintain material integrity and incorporate a modifiable interface surface. Parylene-C was chosen as it is highly inert and exhibits excellent nonfouling properties [22] when exposed to a biological environment due to its resonance stabilized structure and chloride functional group.

#### 3.1.2. Plasma Etching

Parylene-C coated ME materials were etched with O_2_ plasma (March Jupiter II RIE system) for 30 s in order to increase surface charge and roughness [42], thereby introducing a surface conducive to bacterial attachment. O_2_ plasma was used due to its capacity to chemically disrupt the Parylene-C substructure [34]. It should be noted that this process was performed in order to produce a surface to which bacteria would readily adhere; whereas untreated Parylene-C surfaces were not suitable for this study in that they discouraged attachment to a degree where the effects of vibrational loading could not be observed. 

#### 3.1.3. Explanted Sensors

To simulate physiologically relevant conditions, Parylene-C coated ME materials were exposed for 60 days to an *in vivo* environment in age and gender matched, four week old BALB/c mice with an average weight of 30 g at a dorsal site. The animals were housed and used in accordance with protocols approved by the Institutional Animal Care Use Committee at Michigan Technological University. ME strips were explanted with intact surrounding tissue, and subsequently removed. Care was taken to minimize tissue damage during this process. Excised ME materials were fixed and stored in 95% ethanol for less than 24 h.

#### 3.1.4. Poly-L-Lactide (PLLA)

The chemical structure of PLLA does not resist protein adsorption to the same extent as does Parylene-C; in addition to the absence of resonant stabilization, PLLA contains a carbonyl group that interacts readily with some bacterial adhesins [34]. To observe the effects of vibrational loading on a more chemically active surface, plasma etched Parylene-C coated materials were immersed in Bovine Serum Albumen (10% V/V BSA in PBS) and incubated at 37° C for 12 h. A PLLA mixture was generated using 0.2 g PLLA beads (NatureWorks^TM^; grade 6201D) (Minnetonka, MN, USA) dissolved in 4 g chloroform. Following incubation, PLLA was drip coated onto the material surface and cross-linked at 50° C for 3 h. 

#### 3.1.5. Surface Characterization

Contact angle measurements were performed with a Kruss G10 goniometer (Rame-Hart, Inc., Succasunna, NJ, USA) using sessile drop analysis of 2–10 µL deionized H_2_O onto 3 samples. Surface topography was measured using a Nanoscope E (Digital Instruments) Atomic Force Microscopy (AFM) system and a micro-fabricated silicon nitride cantilever tip under constant deflection mode in air over a 5 µm scan area for 3 samples. Software (Digital Instruments) was used to calculate root mean squared roughness, defined as the standard deviation of elevation with respect to the mean of a scanned measured area.

### 3.2. Bacterial Culture

300 μL Brain-Heart infusion (BHI) media (BD 221812) was added to freeze dried *E. coli* (ATCC 25922), *S. aureus* (ATCC 25923), or *S. epidermidis* (ATCC 12228) and agitated. These particular strains were selected based on their clinical relevance, as well as their varying adhesion capacities. 6 mL BHI media was added to bacteria suspension and incubated at 37 °C for 48 h. Following incubation, samples were centrifuged at 1000 rpm for 5 min and supernatant was aspirated off. Subsequently, 5 mL freeze media (10% V/V DMSO in PBS) was added to the resulting pellet, divided into 100 μL aliquots and stored at −20 °C for later use.

Before experimentation, bacterial samples were thawed, added to 10 mL BHI media and incubated at 37° C for 12 h. Prior to vibration, ME materials were submerged in a 1 mL bacterial suspension and subsequently incubated at 37° C for 6 h. Adherent bacteria were stimulated with sub-micron (0.1542 µm) vibrations (158 to 168 kHz) for one hour using a previously developed ME vibrational loading system [33]. 

### 3.3. Colony Staining and Fluorescent Imaging

For all experiments involving fluorescent microscopy, BacLight bacterial viability assay (Invitrogen L7012, Grand Island, NY, USA) with a 1:300 ratio was added to bacterial suspension and incubated at room temperature for 15 min in the dark before imaging. All fluorescent imaging was performed on an Olympus BX51 upright fluorescent microscope.

### 3.4. Statistical Analysis

Images were evaluated (BioQuant) and statistical analysis was performed with a student’s t test (JMP software). Data is expressed as the ±standard error of the mean, and *p*-values less than 0.05 (*p* < 0.05) were considered significant.

## 4. Conclusions

Although Parylene-C and plasma etched Parylene-C coated materials express anti-microbial properties as a result of their surface characteristics, these surface treatments impose only a transient impediment to bacterial colonization. The application of sub-micron vibrational loads serves as both an unimposing and effective measure to significantly reduce bacterial adhesion. Similar detachment trends were observed when bacteria were cultured on a chemically active surface. Sub-micron vibrational loading can serve the dual purpose of acting as a secondary means to deter bacterial adhesion on an anti-microbial surface, as well as a primary means on a chemically active surface. As activation of ME materials is dependent only on externally applied magnetic fields, treatments involving sub-micron vibrations can be applied at any time after deployment of the implant.
